# Dandruff

**Published:** 1873-03

**Authors:** 


### DANDRUFF

Is very readily removed from the scalp by putting a level teaspoonful of Borax in a basin; add three or four tablespoons of warm water, and when well dissolved, rub this into the scalp and roots of the hair with the balls of the fingers; it soon makes a foam like soapsuds, and very effectually removes every particle of grease from the hair and scalp, softens the scales of dandruff, leaving both the hair and scalp perfectly clean; rinse well with warm water, then with cold; wipe the hair well with a soft towel, let it dry for a while, and then dress it; it will be left almost like the softest silk.

A handful of borax in ten gallons of water for washing clothes, which, if let remain in the water all night, will save half the amount of soap, and half the labor of washing.

If pulverized borax is scattered over shelves and other places where cockroaches congregate, it will cause them to disappear speedily.

A teaspoonful of borax, dissolved in two ounces of vinegar, is an excellent wash for ring-worm of the scalp, if the diet consists of fruits and coarse bread for a few days.

It is a mixed, harmless, white powder, which, if applied to the mouths of young children covered with little white patches, corrects the trouble promptly. If made into a wash, it is good for any scaly eruption of the skin.

